# The Antimicrobial Activity of Silver Nanoparticles Biosynthesized Using *Cymbopogon citratus* Against Multidrug-Resistant Bacteria Isolated from an Intensive Care Unit

**DOI:** 10.3390/ph18081120

**Published:** 2025-07-27

**Authors:** Bianca Picinin Gusso, Aline Rosa Almeida, Michael Ramos Nunes, Daniela Becker, Dachamir Hotza, Cleonice Gonçalves da Rosa, Vanessa Valgas dos Santos, Bruna Fernanda da Silva

**Affiliations:** 1 Multi-User Laboratory, Postgraduate Program in Environment and Health, Planalto Catarinense University, Lages 88509-900, SC, Brazil; bianca.p.gusso@gmail.com (B.P.G.); michael.nunes@ifsc.edu.br (M.R.N.); vanessavalgas@uniplaclages.edu.br (V.V.d.S.); brusilvabio@uniplaclages.edu.br (B.F.d.S.); 2 Laboratory of Plasmas, Films, and Surfaces, Santa Catarina State University (UDESC), Joinville 89219-710, SC, Brazil; alinerosaufpr@gmail.com (A.R.A.); daniela.becker@udesc.br (D.B.); 3 Graduate Program in Chemical Engineering (PosENQ), Department of Chemical and Food Engineering (EQA), Federal University of Santa Catarina (UFSC), Florianopolis 88040-900, SC, Brazil; dhotza@gmail.com; 4 Federal Institute of Santa Catarina, Lages 88506-400, SC, Brazil

**Keywords:** antimicrobial activity, disinfectant, nanoparticles, antibiotic-resistant bacteria

## Abstract

**Objective:** This study aimed to evaluate the in vitro efficacy of silver nanoparticles (AgNPs) synthesized by bioreduction using lemongrass (*Cymbopogon citratus*) essential oil against multidrug-resistant (MDR) bacteria isolated from an Intensive Care Unit (ICU). **Methods:** The essential oil was extracted and characterized by gas chromatography–mass spectrometry (GC-MS). Antioxidant activity was assessed using the 2,2-diphenyl-1-picrylhydrazyl (DPPH) radical scavenging assay, the 2,2′-azino-bis (3-ethylbenzothiazoline-6-sulfonic acid) (ABTS) assay, and total phenolic content. AgNPs (3 mM and 6 mM silver nitrate) were characterized by UV-Vis spectroscopy, dynamic light scattering (DLS), zeta potential, transmission electron microscopy (TEM), and Fourier-transform infrared (FTIR) spectroscopy. Bacterial isolates were obtained from ICU surfaces and personal protective equipment (PPE). **Results:** The essential oil presented citral A, citral B, and β-myrcene as major components (97.5% of identified compounds). AgNPs at 3 mM showed smaller size (87 nm), lower Polydispersity Index (0.14), and higher colloidal stability (−23 mV). The 6 mM formulation (147 nm; PDI 0.91; −10 mV) was more effective against a strain of *Enterococcus* spp. resistant to all antibiotics tested. FTIR analysis indicated the presence of O–H, C=O, and C–O groups involved in nanoparticle stabilization. **Discussion:** The higher antimicrobial efficacy of the 6 mM formulation was attributed to the greater availability of active AgNPs. **Conclusions:** The green synthesis of AgNPs using *C. citratus* essential oil proved effective against MDR bacteria and represents a sustainable and promising alternative for microbiological control in healthcare environments.

## 1. Introduction

Antimicrobial resistance (AMR) is considered one of the major threats to global public health [1]. In 2019, an estimated 4.95 million deaths were associated with, and 1.27 million deaths were directly attributable to, bacterial resistance worldwide [2]. Key multidrug-resistant (MDR) pathogens associated with high mortality include *Escherichia coli*, *Staphylococcus aureus*, *Klebsiella pneumoniae*, *Streptococcus pneumoniae*, *Acinetobacter baumannii*, and *Pseudomonas aeruginosa* [2].

The overuse and misuse of antimicrobials in humans, animals, and agriculture is a key driver of the emergence of drug-resistant pathogens. This multifactorial and global problem demands a One Health approach, which recognizes the interdependent relationship between human, animal, plant, and environmental health [1,3].

In this context, the hospital environment, particularly the Intensive Care Unit (ICU), is a high-risk setting for the development of bacterial resistance. This is due to the extensive use of antibiotics, the severity of the patients’ conditions, and the frequency of invasive procedures, all of which increase selective pressure and promote the emergence of resistant microorganisms [4,5]. Within this setting, strengthening infection control measures, such as the rigorous disinfection of surfaces and equipment, is essential to curb the transmission of resistant pathogens. Personal protective equipment (PPE), such as gowns, gloves, and masks, plays a crucial role in preventing cross-contamination in healthcare environments. However, in high-risk settings like ICUs, both inanimate surfaces and PPE serve as critical reservoirs and vectors for multidrug-resistant microorganisms. Consequently, a comprehensive assessment of the microbial burden on both these fomites is crucial to fully understand the dynamics of pathogen transmission and to inform effective infection control strategies [6].

The use of effective products for hospital disinfection is critical to prevent cross-contamination and ensure a safe environment [7]. Consequently, substances with demonstrated antimicrobial activity are being explored for the development of more effective disinfection technologies. Among these, metallic nanoparticles are of particular interest, especially silver nanoparticles (AgNPs), which are renowned for their potent bactericidal and bacteriostatic properties [8].

Although various metallic nanoparticles, such as zinc oxide (ZnO), copper oxide (CuO), and gold (Au), also exhibit antimicrobial activity, AgNPs are particularly promising, demonstrating superior efficacy at lower minimum inhibitory concentrations (MICs) against a wide range of pathogens—including ESKAPE organisms and biofilm-associated bacteria—coupled with a lower propensity for inducing resistance [9,10,11].

Traditionally, AgNPs are produced via physicochemical methods, which often involve hazardous reagents and harsh reaction conditions. In contrast, green synthesis has emerged as an eco-friendly alternative that employs biological entities like plant extracts [12,13], fungi [14], or bacteria [15] as reducing and capping agents. This approach not only minimizes environmental impact but may also enhance the nanoparticles’ biological activity through synergistic effects with the phytochemicals present in the extract [12,13,16]. The antimicrobial power of AgNPs stems from multiple mechanisms, including the disruption of cell membrane integrity, inhibition of DNA replication, generation of reactive oxygen species (ROS), and impairment of essential metabolic pathways [17].

Among these green synthesis resources, the essential oil of *Cymbopogon citratus* (lemongrass) represents an environmentally sustainable and technically feasible approach. This method holds potential for application in disinfectant formulations designed for hospital use [18].

The essential oil of lemongrass is rich in compounds such as citral (geranial and neral) and myrcene, which possess both intrinsic antimicrobial activity and excellent reducing potential for AgNP synthesis. Several studies have successfully reported the synthesis of AgNPs using *C. citratus* and have evaluated their efficacy against standard reference bacterial strains [18,19,20,21,22]. However, there is a lack of research evaluating the potential of these specific nanoparticles against clinically relevant, multidrug-resistant isolates obtained from high-risk environments, such as ICUs. The efficacy against these “wild-type” strains, which have evolved complex resistance mechanisms in a real-world setting, cannot be directly extrapolated from data on laboratory strains. Therefore, investigations targeting clinically significant pathogens are crucial to validate the translational potential of these nanotechnologies for hospital infection control.

Therefore, the aim of this study was to evaluate the in vitro antimicrobial activity of AgNPs, synthesized via bioreduction using *C. citratus* essential oil, against multidrug-resistant bacteria isolated from surfaces and personal protective equipment (PPE) in an Intensive Care Unit (ICU).

## 2. Results and Discussion

### 2.1. Chemical Characterization, Antioxidant Activity, and Total Phenolic Content of Cymbopogon citratus Essential Oil

Essential oils (EOs) are complex mixtures of secondary metabolites produced by plants. Consequently, their chemical composition is known to vary depending on several factors, including environmental conditions, the plant part used for extraction, and the harvesting season [23]. In this study, the major compounds identified in the *C. citratus* essential oil were geranial (citral A), accounting for 41.6% of the total composition, followed by neral (citral B) (31.8%) and β-myrcene (19.3%), as detailed in Table 1.

Citral is an acyclic monoterpene aldehyde with a characteristic lemon-like aroma, comprising a mixture of two geometric isomers: geranial (trans-citral or citral A) and neral (cis-citral or citral B). It is the primary component of essential oils derived from *Cymbopogon* species [24].

The reported biological properties of lemongrass essential oil include antioxidant, anti-inflammatory, antimicrobial, and antidiabetic activities [23]. Furthermore, citral itself exhibits antiparasitic, allelopathic, and mosquito-repellent properties. Citral is a highly valued monoterpene in the flavor, fragrance, cosmetic, and pharmaceutical industries. It serves as a key precursor for the synthesis of vitamin A, β-ionones, and other specialty chemicals [24].

As shown in Table 2, the lemongrass essential oil demonstrated antioxidant activity, evaluated by its capacity to scavenge the 2,2-diphenyl-1-picrylhydrazyl (DPPH•) and 2,2′-azino-bis (3-ethylbenzothiazoline-6-sulfonic acid) (ABTS•+) radicals. This activity is consistent with its total phenolic content. The essential oil exhibited a higher scavenging capacity against the ABTS•+ radical compared to the DPPH• radical. This result highlights the importance of employing multiple methods to obtain a comprehensive assessment of antioxidant capacity [25].

*Cymbopogon citratus*, belonging to the Poaceae family, is a plant rich in essential oil and also contains a variety of phytochemicals, including flavonoids, alkaloids, tannins, carbohydrates, steroids, and phytosterols [26]. Since essential oils are complex mixtures of multiple compounds, their overall antioxidant efficacy cannot be attributed to a single mechanism but rather to the synergistic or additive effects of their constituents [27].

However, the overall antioxidant capacity is often linked to major constituents, particularly phenolic compounds, which act as primary antioxidants by scavenging free radicals [28]. The antioxidant action of phenolics can interfere with the three stages of lipid peroxidation: initiation, propagation, and termination. Their hydroxyl groups (-OH) can donate hydrogen atoms to inactivate free radicals, such as peroxyl and alkoxyl radicals. The redox properties of phenolic compounds stem from their ability to act as reducing agents, hydrogen donors, and singlet oxygen quenchers, making them potent antioxidants that prevent the oxidation of other molecules [28].

### 2.2. Synthesis and Physicochemical Characterization of Silver Nanoparticles

**Figure 1:**
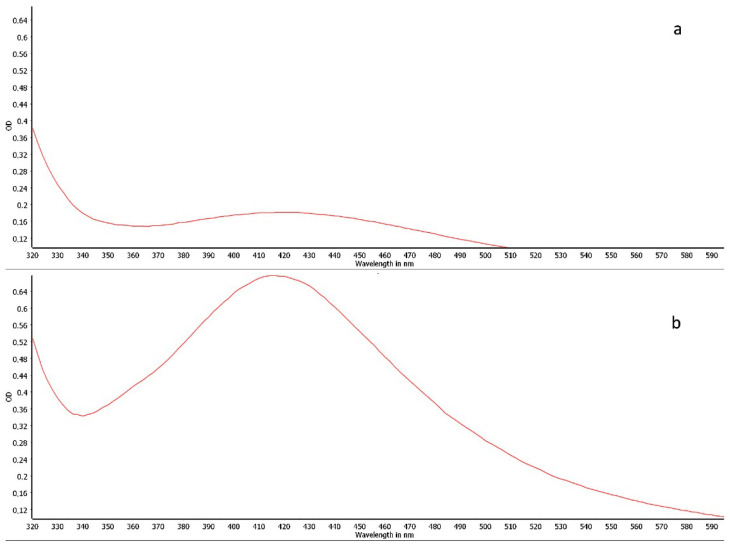
UV–Vis spectra of AgNPs synthesized with *Cymbopogon citratus* essential oil using AgNO_3_ at (**a**) 3 mM and (**b**) 6 mM.

The formation of silver nanoparticles (AgNPs) was monitored by UV-Vis spectroscopy. The successful synthesis was confirmed by the appearance of a characteristic absorption band in the 400–450 nm region, which is attributed to the Surface Plasmon Resonance (SPR) phenomenon of AgNPs (Figure 1a,b).

A visual color change is the initial qualitative indicator of AgNP synthesis. In this study, the reduction of silver ions (Ag^+^) was evidenced by a shift in the reaction solution’s color from colorless to a pale orange-brown. This color change indicates that the bioreduction of AgNO_3_ was successfully mediated by the essential oil, leading to the formation of nanoparticles [29,30].

Due to its significant phenolic content and demonstrated antioxidant capacity, *C. citratus* essential oil acts as a potent reducing and capping agent. It facilitates the reduction of silver ions (Ag^+^) to metallic silver (Ag^0^), which then undergoes nucleation and growth to form stable silver nanoparticles [18,29,31].

In addition to UV-Vis spectroscopy, the formation and morphology of the AgNPs were further confirmed by transmission electron microscopy (TEM), a cornerstone technique for nanoparticle characterization.

**Figure 2:**
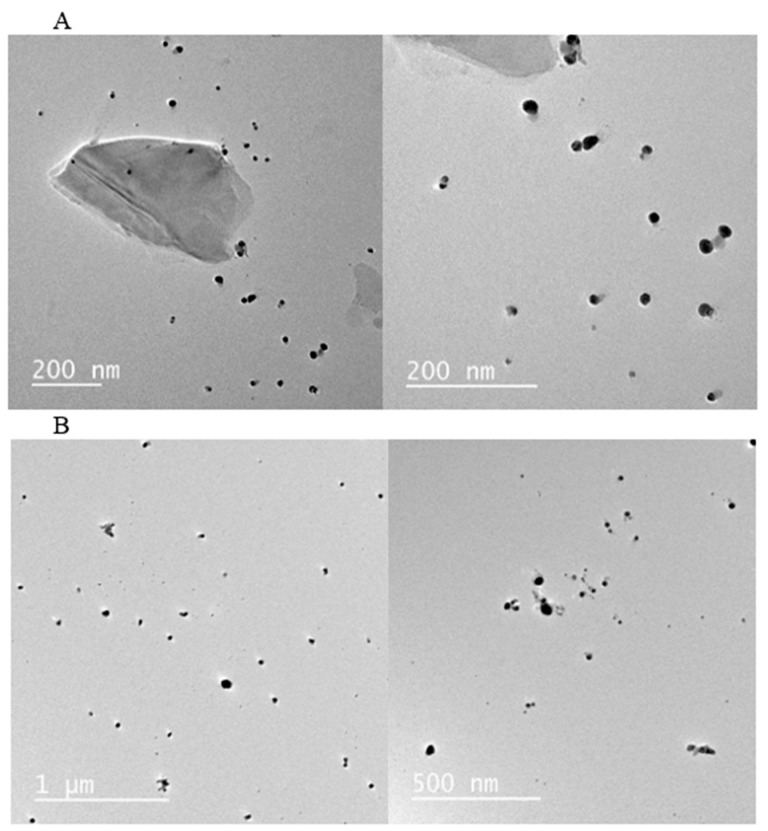
Transmission electron microscopy (TEM) micrographs of silver nanoparticles (AgNPs) synthesized using *Cymbopogon citratus* essential oil. The images show nanoparticles produced with different initial concentrations of the AgNO_3_ precursor: (**A**) 3 mM and (**B**) 6 mM.

In the present study, the TEM micrographs revealed that the synthesized AgNPs were predominantly spherical in shape with a smooth surface (Figure 2A,B). This morphology is characteristic of silver nanoparticles and is consistent with previous reports [32,33].

As presented in Table 3, the AgNPs synthesized with 3 mM AgNO_3_ exhibited superior physicochemical properties compared to those synthesized with 6 mM AgNO_3_. The 3 mM formulation yielded smaller nanoparticles with a mean hydrodynamic diameter of 87.0 ± 1.1 nm and showed greater homogeneity, as indicated by a low Polydispersity Index (PDI) of 0.14 ± 0.006. A PDI value below 0.2 is typically associated with a narrow and uniform size distribution. Furthermore, these nanoparticles displayed a zeta potential of −23.0 ± 0.4 mV, suggesting moderate colloidal stability maintained by electrostatic repulsion between particles. Values approaching ± 30 mV are generally considered indicative of good stability.

The final size of the synthesized silver nanoparticles is dependent on both the initial AgNO_3_ concentration and the reducing potential of the essential oil. In green synthesis, the essential oil acts not only as a reducing agent but also as a stabilizing (or capping) agent, where its phytochemicals coat the surface of the metallic nanoparticles, preventing aggregation [34].

Zeta potential values approaching or exceeding ±30 mV are generally indicative of a stable colloidal dispersion, as the high surface charge promotes strong electrostatic repulsion between particles, thus preventing aggregation [33,34]. The Polydispersity Index (PDI) is a key parameter that measures the uniformity of the particle size distribution. Low PDI values (typically <0.3) signify a monodisperse and homogeneous sample, which is crucial for the long-term stability of the colloidal suspension and prevents the formation of aggregates or precipitates [29].

Nanoparticles exhibit unique properties that are distinct from both individual atoms and their bulk metallic counterparts. In this context, their optical properties, such as the color and the position of the UV-Vis absorption maximum, are governed by the Surface Plasmon Resonance (SPR). The SPR is highly sensitive to the nanoparticle’s size, shape, and the surrounding dielectric medium. An increase in particle size typically causes a red-shift (a shift to longer wavelengths) in the absorption maximum of the SPR band. This phenomenon was observed in our study, where the 6 mM AgNP formulation, which produced larger particles (Table 3), displayed an absorption maximum at a longer wavelength compared to the 3 mM formulation (Figure 2B vs. Figure 1a) [35].

**Figure 3:**
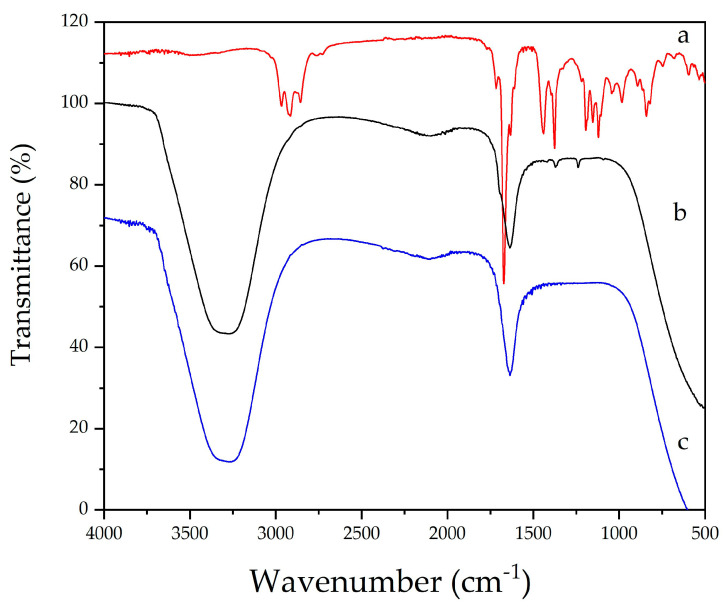
FTIR spectra of silver nanoparticles (AgNPs) and *Cymbopogon citratus* essential oil: (a) *C. citratus* essential oil; (b) AgNPs synthesized with 3 mM silver nitrate precursor; and (c) AgNPs synthesized with 6 mM silver nitrate precursor.

The stabilization and formation of silver nanoparticles (AgNPs) were investigated by Fourier-transform infrared (FTIR) spectroscopy, as shown in Figure 3. The spectra obtained correspond to the essential oil of *C. citratus* (a) and AgNPs synthesized with 3 mM (b) and 6 mM (c) of silver nitrate.

The FTIR spectrum of the essential oil (a) revealed characteristic bands attributed to functional groups with well-known reducing and stabilizing potential. The broad band observed at ~3400 cm^−1^ corresponds to the O–H stretching vibrations of alcohols and phenols, while the bands at ~2920 and ~2850 cm^−1^ are associated with the C–H stretching of aliphatic chains (–CH_3_ and –CH_2_). The intense band near 1640–1600 cm^−1^ is assigned to conjugated C=O stretching or C=C stretching of aromatic and olefinic compounds present in citral. Signals between 1400 and 1000 cm^−1^ may be attributed to C–O and C–N vibrations, typical of ethers and phenolic compounds, while bands around ~700–600 cm^−1^ indicate possible out-of-plane bending of aromatic C–H bonds [31].

In the spectra of AgNPs synthesized in aqueous medium (b and c), the presence of O–H (~3400 cm^−1^) and C=O (~1640 cm^−1^) bands was observed, along with slight shifts in their positions, indicating direct interaction of these groups with the nanoparticle surface. These changes confirm that the bioactive compounds in the essential oil acted both in the reduction of Ag^+^ to Ag^0^ and in the stabilization of the metallic particles, forming an organic layer around the AgNPs [18,31].

A comparison between the 3 mM and 6 mM AgNO_3_ concentrations revealed that the formulation with the higher concentration (profile c) exhibited more pronounced changes in the bands, suggesting a greater consumption of functional groups from the essential oil during the redox process. This behavior is consistent with the literature, which associates the intensity of phytochemical–nanoparticle surface interactions with the efficiency of synthesis and colloidal stability of the resulting system [18].

Thus, the FTIR results support the findings obtained from other analytical techniques, confirming the simultaneous role of the essential oil as both a reducing and capping agent. The presence of compounds such as citral (geranial and neral) and β-myrcene was essential for the successful green synthesis of AgNPs, contributing to their stabilization through bioactive functional groups and preventing colloidal aggregation.

### 2.3. Microbiological Analysis, Isolate Selection, and Antimicrobial Susceptibility

A total of 54 samples were collected from surfaces within an ICU, of which 88.6% (*n* = 39) yielded bacterial growth. Among the positive samples, 69.2% (*n* = 27) were identified as Gram-positive bacteria, while 30.8% (*n* = 12) were Gram-negative. Surfaces with lower physical contact, such as cardiac monitors, computer desks, and doorknobs, showed less contamination. In contrast, high-touch surfaces and items frequently handled by healthcare professionals and patients, including stethoscopes, scrubs, and infusion pumps, yielded a higher number of positive isolates.

The isolation of *Staphylococcus epidermidis* (14 isolates), followed by *Staphylococcus saprophyticus* (5 isolates), indicates a predominance of Gram-positive bacteria commonly associated with the human skin microbiota. Studies show that *S. epidermidis*, although often considered a contaminant, is a significant opportunistic pathogen, particularly in immunocompromised patients or those with invasive devices, due to its ability to form biofilms and its growing antimicrobial resistance [36]. Regarding *Staphylococcus saprophyticus*, while its role as a causative agent of nosocomial infections is uncommon, it is important to note that this pathogen has the ability to organize into biofilms, which can result in antimicrobial resistance [37].

The detection of *Enterococcus* spp. (four isolates), *Enterobacter* spp. (four isolates), and *Pseudomonas aeruginosa* (three isolates) on frequently used ICU surfaces highlights concerning environmental contamination with pathogens associated with nosocomial infections.

Although *Enterococcus* spp. are part of the human gut microbiota, they are widely recognized as a major agent of hospital-acquired infections. Similarly, *Enterobacter* spp. are considered one of the main Gram-negative bacilli isolated in ICUs, with the potential to develop resistance mechanisms, such as the production of extended-spectrum β-lactamases, which compromises the efficacy of various antimicrobial classes. Their presence on hospital surfaces, often related to the use of invasive devices, reinforces the risk of cross-transmission and secondary infection [38].

*Pseudomonas aeruginosa*, in turn, is a pathogen notoriously associated with mechanical ventilation, catheters, and humid environments, with a high capacity for survival under adverse conditions, as well as for forming biofilms, which complicates its eradication. Furthermore, its multidrug-resistant profile has been a cause for concern in hospital environments around the globe [39].

Thus, the presence of these bacteria on frequently used ICU surfaces demonstrates the need for disinfection protocols and reinforces the use of sanitizers with microbicidal potential, in order to contain the dissemination of pathogens, including those that are less common but potentially dangerous [39]. These findings point to the importance of environmental control as a strategic component in combating healthcare-associated infections.

Of the bacteria that showed resistance to all antibiotics, only *Enterococcus faecalis* was resistant to the tested antimicrobials. *Enterococcus*, particularly the species *E. faecalis* and *E. faecium*, plays a relevant role in nosocomial infections worldwide, being responsible for 10% of cases, with a higher incidence in ICU environments [40]. This is due to its high adaptability to the hospital environment, surviving for long periods on inanimate surfaces and resisting adverse conditions, which facilitates its dissemination [41,42].

Antimicrobial susceptibility testing was performed using the disk diffusion method on Mueller–Hinton agar, following the CLSI M100 guidelines [43]. A panel of 26 antibiotics representing various classes commonly used in clinical practice—particularly in intensive care settings—was employed. The analysis revealed heterogeneous resistance and sensitivity profiles among the isolates. Staphylococcus epidermidis, the most frequently recovered species, exhibited sensitivity to several antimicrobials, including amikacin, gentamicin, and ciprofloxacin; however, certain isolates displayed resistance to up to seven antibiotics, indicating a multidrug-resistant (MDR) phenotype. *Enterobacter* spp. and *Pseudomonas aeruginosa* demonstrated broad resistance, notably to extended-spectrum β-lactams (e.g., ceftriaxone and meropenem), fluoroquinolones, and aminoglycosides. Particularly concerning were cases involving *Enterococcus* spp., with one isolate showing complete resistance to all tested antimicrobials—characterizing pan-resistance. Resistance to three or more antimicrobial classes was observed in several isolates, fulfilling the MDR definition [44]. These findings underscore the clinical and epidemiological importance of continuous surveillance and reinforce the urgent need for effective infection control protocols and antimicrobial stewardship strategies, especially in high-complexity environments such as ICUs.

Mortality associated with nosocomial infections by *Enterococcus* ssp., especially in cases of bacteremia, can reach 20%, but these values can exceed 30% in cases of vancomycin-resistant strains [45,46]. Currently, vancomycin-resistant *Enterococcus* ssp. represents one of the main challenges in hospital infections due to its high colonization capacity, environmental persistence, and antimicrobial resistance, especially in high-complexity environments such as ICUs. Vancomycin resistance significantly limits the therapeutic options for treating *Enterococcus* ssp. infections, requiring the use of antimicrobials such as linezolid or daptomycin, which may have higher toxicity or cost.

### 2.4. In Vitro Antimicrobial Activity of Silver Nanoparticles Synthesized with Cymbopogon citratus Essential Oil

To evaluate their antimicrobial efficacy, the two synthesized AgNP formulations (derived from 3 mM and 6 mM AgNO_3_ precursors) were tested against the multidrug-resistant *Enterococcus faecalis* isolate at three different bacterial concentrations: 4.5 × 10^5^ CFU/mL, 3.0 × 10^5^ CFU/mL, and 1.5 × 10^5^ CFU/mL.

The results revealed that the AgNP formulation derived from a 3 mM precursor exhibited limited antimicrobial activity against the highest bacterial load (4.5 × 10^5^ CFU), with counts remaining above 100,000 CFU. However, against lower bacterial concentrations of 3.0 × 10^5^ CFU and 1.5 × 10^5^ CFU, this formulation showed moderate and restricted growth, with 214 CFU and 173 CFU, respectively.

In contrast, the AgNPs synthesized with a 6 mM precursor demonstrated significantly higher efficacy. Against bacterial loads of 4.5 × 10^5^ CFU, 3.0 × 10^5^ CFU, and 1.5 × 10^5^ CFU, the outcomes were moderate growth (232 CFU), restricted growth (61 CFU), and a complete absence of bacterial growth, respectively.

Thus, the data indicate that the antimicrobial activity of the AgNPs is directly related to their concentration, revealing a dose-dependent profile. The 3 mM AgNP formulation was insufficient to significantly inhibit bacterial growth at high microbial loads (4.5 × 10^5^ CFU), with counts exceeding 100,000 CFU, indicating low efficacy under this condition. However, as the initial CFU count was decreased (to 3.0 × 10^5^ and 1.5 × 10^5^ CFU), a greater inhibitory effect was observed, with colony counts reduced to 214 and 173 CFU, respectively, demonstrating a partial antimicrobial effect.

Conversely, the 6 mM AgNP formulation showed substantially greater efficacy. Even against a high bacterial concentration (4.5 × 10^5^ CFU), growth was limited to 232 CFU. At a load of 3.0 × 10^5^ CFU, the count was reduced to 61 CFU, and at 1.5 × 10^5^ CFU, complete inhibition of growth was observed, corroborating a potentiated bactericidal effect with an increased nanoparticle dose.

These findings are in agreement with previous studies demonstrating that both the concentration and size of AgNPs are critical determinants of their antimicrobial efficacy. According to Lara et al. (2011) [47], higher concentrations of AgNPs enhance their interaction with the bacterial cell wall, leading to membrane disruption and the leakage of intracellular contents. Furthermore, Holubnycha et al. (2024) [48] highlight that an increased dose can overcome bacterial defense mechanisms, such as biofilm formation.

Additionally, studies like that of Ershov et al. (2024) [49] indicate that not only concentration but also particle size influences antimicrobial efficacy, with smaller particles generally being more active due to their larger surface-area-to-volume ratio. According to Hussien et al. (2024) [50], the antimicrobial activity can be attributed to multiple biochemical and molecular mechanisms triggered by the silver nanoparticles synthesized with *C. citratus* essential oil. AgNPs are known to disrupt bacterial cell membranes, increasing permeability and leading to leakage of intracellular contents. In addition, they release Ag^+^ ions, which can bind to thiol groups in vital bacterial enzymes, impairing metabolic functions. Moreover, AgNPs can induce the generation of reactive oxygen species (ROS), causing oxidative stress, lipid peroxidation, protein denaturation, and DNA fragmentation.

In this research, the nanoparticles were synthesized in the presence of *C. citratus* essential oil, which acts as both a reducing and capping agent. This essential oil has been extensively studied for its antimicrobial properties, demonstrating in vitro efficacy against various microorganisms, including Gram-positive and Gram-negative bacteria such as *Staphylococcus aureus*, *Bacillus subtilis*, *Pseudomonas aeruginosa*, and multidrug-resistant strains of *Acinetobacter baumannii* [51].

The antimicrobial potential of *C. citratus* essential oil is primarily attributed to the presence of bioactive compounds like citral and other monoterpenes, such as α-terpineol and myrcene, which act synergistically to enhance its bactericidal and fungicidal effects. One of the proposed mechanisms is the ability of these compounds to compromise the integrity of the microbial cell membrane, promoting structural disorganization, loss of intracellular contents, and subsequent cell death. Moreover, the oil has been shown to inhibit the formation and stability of biofilms, a relevant aspect in treating persistent infections [52].

Therefore, the results of this study reinforce the potential of AgNPs synthesized with *C. citratus* essential oil as promising sanitizing agents, particularly for healthcare environments. The nanoparticles demonstrated significant in vitro antimicrobial activity against multidrug-resistant strains of *Enterococcus* sp. isolated from an ICU. These findings suggest their applicability in disinfection strategies aimed at controlling the spread of resistant pathogens in critical care settings.

## 3. Materials and Methods

### 3.1. Materials

The following materials used in this study were all purchased from Sigma-Aldrich (Saint Louis, MO, USA): silver nitrate (AgNO_3_), acetone, DPPH (2,2-diphenyl-1-picrylhydrazyl), ABTS (2,2′-azino-bis (3-ethylbenzothiazoline-6-sulfonic acid)), and Trolox (6-hydroxy-2,5,7,8-tetramethylchroman-2-carboxylic acid). Hexane (analytical grade) and sodium hydroxide (NaOH) were obtained from Merck (Darmstadt, Germany).

Culture media used for bacterial isolation and identification included Blood Agar and MacConkey Agar (Oxoid, Basingstoke, UK), Brain Heart Infusion (BHI) broth and agar (Himedia, Thane, India), and Stuart transport medium (Oxoid, Basingstoke, UK). Biochemical identification systems included Bactray-3 (Quibasa-Bioclin, Belo Horizonte, Brazil) and modified Rugai medium (Himedia, Mumbai, India). Other identification reagents, such as catalase (Synth, Diadema, Brazil), coagulase plasma (Sigma-Aldrich, St. Louis, MO, USA), bile esculin agar (Himedia, Mumbai, India), and novobiocin and optochin disks (Oxoid, Basingstoke, UK), were also used. All other chemicals and solvents were of analytical grade.

### 3.2. Essential Oil Extraction, Chemical Characterization, Antioxidant Activity, and Total Phenolic Content of Cymbopogon citratus

The essential oil (EO) of lemongrass (*C. citratus*) was extracted from approximately 1 kg of fresh leaves by steam distillation using a Clevenger-type apparatus for 6 h. The chemical composition of the EO was characterized by gas chromatography–mass spectrometry (GC-MS) using a GCMS-QP2010 system (Shimadzu, Kyoto, Japan).

A ZB-5MS capillary column (30 m × 0.25 mm i.d., 0.25 µm film thickness) was used. The injector temperature was set at 250 °C, and helium was used as the carrier gas at a constant flow rate of 1.0 mL/min. The oven temperature program was set as follows: an initial temperature of 60 °C held for 4 min, followed by a ramp to 210 °C, where it was held for 6 min, for a total run time of 35 min. The oil samples were diluted 1:200 (*v*/*v*) in chromatographic-grade hexane prior to injection.

The relative percentage of each component was calculated by the area normalization method from the total ion chromatogram (TIC), where the total area represented the sum of all eluted peaks (100%). The retention indices (RIs) were calculated according to the van den Dool and Kratz (1963) [53] equation using a homologous series of n-alkanes (C7–C30) as standards, analyzed under the same chromatographic conditions as the samples. Component identification was based on a dual approach: comparison of their mass spectra with those from the NIST-05 library and comparison of their calculated RIs with those reported in the literature (e.g., NIST WebBook, GMD databases).

The total phenolic content (TPC) of the essential oil was quantified based on the methodology proposed by Swain and Hillis (1959) [54], with modifications as described by Nunes et al. (2018) [32]. A standard curve was constructed using geranial, and the results were expressed as milligrams of Geranial Equivalents per milliliter of oil (mg GEs/mL).

The antioxidant activity of the EO was evaluated using two methods: the DPPH• radical scavenging assay and the ABTS•+ radical cation scavenging assay.

The DPPH• radical scavenging activity was assessed according to the method of Brand-Williams, Cuvelier, and Berset (1995) [55]. Briefly, 150 µL of the EO was mixed with 2850 µL of a 0.1 mM DPPH• solution (dissolved in methanol). The mixture was incubated at 22 ± 2 °C in the dark for 24 h. The absorbance was then measured at 515 nm using a Labman 752D spectrophotometer (Nanchang, China). A calibration curve was prepared using Trolox, and the antioxidant activity was expressed as milligrams of Trolox Equivalents per milliliter of oil (mg TEs/mL).

The ABTS•+ radical scavenging activity was determined following the method of Re et al. (1999) [56], with modifications. Briefly, 30 µL of the EO was added to 3000 µL of the ABTS•+ radical solution and homogenized using a vortex mixer. After a 6 min incubation period at room temperature, the absorbance was measured at 734 nm using a 752D spectrophotometer (Labman, Nanchang, China). The results were also expressed as mg of Trolox Equivalents per milliliter of essential oil (mg TEs/mL).

### 3.3. Synthesis and Characterization of AgNPs via Bioreduction with Cymbopogon citratus Essential Oil

AgNPs were synthesized via bioreduction using lemongrass essential oil, following the methodology described by Nunes et al. (2018) [32] and De Melo et al. (2020) [34], with minor modifications. Briefly, 30 mL of an aqueous silver nitrate (AgNO_3_) solution (at concentrations of 3 × 10^−4^ mol/L and 6 × 10^−4^ mol/L) was reduced in the presence of 3 mL of *C. citratus* essential oil previously diluted 1:170 (*v*/*v*) in acetone. The pH of the resulting suspension was adjusted to 9.0 with 0.1 M NaOH, and the mixture was homogenized for 30 min on a magnetic stirrer at 100 °C.

The formation of AgNPs was preliminarily confirmed by UV–Visible (UV-Vis) spectroscopy. The absorption spectra were recorded using a SPECTROstar Nano spectrophotometer (BMG LABTECH, Ortenberg, Germany) in the wavelength range of 200 to 1000 nm.

The mean hydrodynamic diameter (Z-average, nm), Polydispersity Index (PDI), and zeta potential (ζ, mV) of the synthesized AgNPs were determined by dynamic light scattering (DLS) using a Zetasizer Nano instrument (Malvern Panalytical, Worcestershire, UK). Prior to analysis, samples were appropriately diluted in Milli-Q filtered water. Measurements were performed at 25 °C with a scattering angle of 173°. For zeta potential measurements, the samples were placed in a disposable folded capillary cell (DTS1070).

The morphology and size distribution of the AgNPs were assessed by transmission electron microscopy (TEM) using a JEOL JEM-1011 microscope (JEOL Ltd., Tokyo, Japan) operating at an acceleration voltage of 70 kV. The average particle size and distribution were determined by measuring at least 100 individual nanoparticles from the TEM micrographs using ImageJ software Version 1.54p (National Institutes of Health, USA).

To confirm the stabilization of silver nanoparticles (AgNPs) by the functional groups present in the essential oil, Fourier-transform infrared (FTIR) spectroscopy was performed using an IR Spirit spectrometer (Shimadzu, Japan) equipped with an attenuated total reflectance (ATR) accessory. Spectra were acquired in the range of 4000 to 500 cm^−1^, with a resolution of 4 cm^−1^ and 40 scans accumulated per sample.

### 3.4. Bacterial Isolation for Antimicrobial Efficacy Testing

Samples were collected from inanimate surfaces and personal protective equipment (PPE) within a general Intensive Care Unit (ICU) of a hospital located in the Serra Catarinense region (Santa Catarina, Brazil). Swabs pre-moistened with buffered peptone water were rubbed in a zig-zag pattern over high-touch surfaces—including equipment and furniture—frequently contacted by patients and healthcare professionals. Samples were also collected from the PPE (gloves, scrubs, and footwear) of staff present in the ICU at the time of sampling.

Following collection, the swabs were placed into Stuart transport medium and then inoculated into Brain Heart Infusion (BHI) enrichment broth, followed by incubation at 37 °C for 24 h. Subsequently, the enriched cultures were streaked onto Blood Agar (incubated under microaerophilic conditions) and MacConkey Agar plates, which were then incubated at 37 °C for an additional 24 h.

Resulting bacterial colonies were subjected to Gram staining for preliminary identification. Gram-negative isolates were identified using the Bactray-3 biochemical system, supplemented with modified Rugai medium. Gram-positive isolates were presumptively identified based on their hemolysis patterns on Blood Agar, followed by standard biochemical tests, including catalase, coagulase, bile esculin hydrolysis, optochin, and novobiocin susceptibility tests.

Antimicrobial susceptibility testing was performed by the disk diffusion method on Mueller–Hinton agar, following CLSI M100 guidelines [43]. The panel included 26 antibiotics, selected to represent a wide range of antimicrobial classes commonly used in clinical practice, particularly in critical care settings. The agents tested were amikacin, amoxicillin, azithromycin, cefazolin, cephalexin, cefoxitin, cefepime, ceftazidime, cefalotin, ceftriaxone, clindamycin, ciprofloxacin, erythromycin, ertapenem, levofloxacin, linezolid, gentamicin, imipenem, novobiocin, nitrofurantoin, norfloxacin, meropenem, sulfamethoxazole–trimethoprim, tobramycin, and piperacillin–tazobactam. The inhibition zones were measured and interpreted according to CLSI or EUCAST criteria, when applicable. The multidrug-resistant (MDR) profile of the isolates was defined based on resistance to three or more antimicrobial classes, as proposed by Magiorakos [44].

### 3.5. Evaluation of the Antimicrobial Activity of AgNPs

To assess the antimicrobial activity of the AgNPs, bacterial suspensions of the selected Enterococcus sp. isolate were prepared at initial concentrations of 4.5 × 10^5^ CFU, 3.0 × 10^5^ CFU, and 1.5 × 10^5^ CFU. These suspensions were then exposed, in triplicate, to the two AgNP formulations synthesized (from 3 mM and 6 mM AgNO_3_ precursors), resulting in final bacterial concentrations of 2.25 × 10^5^ CFU/mL, 1.5 × 10^5^ CFU/mL, and 7.5 × 10^4^ CFU/mL, respectively. The viability of the bacteria after exposure was quantified using the pour plate technique. The plates were incubated at 37 °C for 24 h, after which bacterial growth was assessed by colony counting. Positive controls consisted of the bacterial suspensions without the addition of AgNPs. Negative controls (sterility controls) consisted of the AgNP suspensions plated alone, without bacteria, to ensure their sterility.

### 3.6. Data Analysis

Data from the bacterial isolation survey in the hospital were analyzed descriptively and are presented as absolute frequencies (n) and percentages (%). Data from the nanoparticle synthesis and characterization, as well as the antimicrobial activity assays, are expressed as the mean ± standard deviation (SD) of three independent experiments (*n* = 3). Statistical analysis was performed using one-way analysis of variance (ANOVA), followed by Tukey’s post hoc test for multiple comparisons. A significance level of *p* < 0.05 was adopted for all analyses. All statistical tests were conducted using STATISTICA software (version 7.0; StatSoft Inc., Tulsa, OK, USA) [57].

## 4. Conclusions

This study demonstrated the in vitro efficacy of silver nanoparticles (AgNPs), synthesized via bioreduction with *Cymbopogon citratus* (lemongrass) essential oil, against a multidrug-resistant *Enterococcus* sp. isolate recovered from a general ICU environment. The microbiological survey of PPE and inanimate surfaces in the ICU revealed significant bacterial contamination, with 88.6% of the 48 collected samples yielding bacterial growth. Gram-positive bacteria were predominant (69.2%), followed by Gram-negative bacteria (30.8%). Key pathogens identified included *Staphylococcus epidermidis*, *Staphylococcus saprophyticus*, *Enterobacter* spp., *Enterococcus* spp., *Pseudomonas aeruginosa*, and *Escherichia coli*. Notably, an *Enterococcus* sp. isolate resistant to all tested antibiotics was detected and selected for this study. The AgNPs produced via bioreduction with lemongrass essential oil were predominantly spherical with a smooth surface. Both formulations (synthesized from 3 mM and 6 mM precursors) showed good homogeneity, with a mean particle size ranging from 87 to 147 nm. The in vitro antimicrobial assays revealed a clear dose-dependent effect: higher concentrations of AgNPs resulted in greater inhibition of *Enterococcus* sp. growth. These findings suggest that the development of sanitizing agents based on these green-synthesized AgNPs is a viable alternative for the disinfection of surfaces and equipment in hospital units. Such an approach could serve as a valuable tool in the prevention and control of bacterial resistance and healthcare-associated infections.

## Figures and Tables

**Table 1:** Chemical composition of *Cymbopogon citratus* essential oil identified by gas chromatography–mass spectrometry (GC-MS).

	RIC	RIL	Composition (%)
Geranial	1268	1271	41.6
Neral	1239	1240	31.8
β-Mirceno	989	989	19.3
Geraniol	1250	1249	2.3
6-Methyl-5-heptene-2-one	984	986	1.7
2-Undecanone	1292	1292	0.7
Total Identified			97.5

RIC = retention index calculated; RIL = retention index from the literature; Composition (%) = percentage of compounds in essential oil.

**Table 2:** Antioxidant activity and total phenolic content (TPC) of *Cymbopogon citratus* essential oil.

Sample	DPPH Assay (µg TEs/mL)	ABTS Assay (µg TEs/mL)	TPC (mg GEs/mL)
Lemongrass EO	282.0 ± 5.6	456.6 ± 11.2	1.2 ± 0.1

Data are presented as mean ± standard deviation (*n* = 3). DPPH: 2,2-diphenyl-1-picrylhydrazyl radical scavenging assay. ABTS: 2,2′-azino-bis (3-ethylbenzothiazoline-6-sulfonic acid) radical scavenging assay. TPC: total phenolic content.

**Table 3:** Hydrodynamic diameter (Z-average), Polydispersity Index (PDI), and zeta potential of AgNPs synthesized with *Cymbopogon citratus* essential oil.

Sample	Z-Average (nm)	PDI	Zeta Potential (mV)
AgNPs (3 mM precursor)	87.0 ± 1.1 ^b^	0.14 ± 0.006 ^b^	−23.0 ± 0.4 ^a^
AgNPs (6 mM precursor)	147.3 ± 1.5 ^a^	0.91 ± 0.006 ^a^	−10.4 ± 0.4 ^b^

Data are presented as mean ± standard deviation (*n* = 3). The precursor concentration refers to the initial AgNO_3_ concentration used during synthesis. Different letters in the same column indicate a significant difference when analyzed by Tukey’s test (*p* < 0.05).

## Data Availability

The original contributions presented in the study are included in the article; further inquiries can be directed to the corresponding author.
